# Kaat koort: Study protocol for a pragmatic randomized controlled trial of a multifactorial, multidisciplinary Aboriginal Health Practitioner-led Aboriginal dementia prevention intervention

**DOI:** 10.1016/j.conctc.2025.101457

**Published:** 2025-02-12

**Authors:** Carrington CJ. Shepherd, Melissa A. Dunham, Lina Gubhaju, Karen E. Lamb, Digsu N. Koye, Phoebe Fitzpatrick, Emily Banks, Kaarin J. Anstey, Melinda Carrington, Daniel McAullay, Ofra Kalter-Leibovici, Grace Joshy, Lesley Nelson, Jason Agostino, Ellie Paige, Kathleen Abu-Saad, Elise Alexander, Rona MacNiven, Kelsey Griffen, Fiona Collins, Salena Linforth-Milham, Dolores Gilbert, Cindy Prior, Sadia Rind, Richelle Douglas, Sandra Eades

**Affiliations:** a Curtin Medical School, Curtin University, Kent Street, 6102, Bentley, Australia; b Ngangk Yira Institute, Murdoch University, South Street, 6150, Murdoch, Australia; c The Kids Research Institute Australia, 15 Hospital Ave, Nedlands, 6009, Australia; d Centre for Epidemiology and Biostatistics, Melbourne School of Population and Global Health, The University of Melbourne, 207-221 Bouverie Street, 3010, Melbourne, Australia; e Methods and Implementation Support for Clinical Health (MISCH) Research Hub, Faculty of Medicine, Dentistry and Health Sciences, University of Melbourne, 161 Barry St, 3010, Carlton, Australia; f National Centre for Epidemiology and Population Health, Australian National University, 62 Mills Rd, 2601, Acton, Australia; g School of Psychology, University of New South Wales, Mathews Building, 2052, Kensington, Australia; h Neuroscience Research Australia (NeuRA), 139 Barker St, Randwick, 2031, Australia; i UNSW Ageing Futures Institute, 2052, Kensington, Australia; j Baker Heart and Diabetes Institute, 75 Commercial Rd, 3004, Melbourne, Australia; k Baker Department of Cardiometabolic Health, The University of Melbourne, Gratton St, 3010, Parkville, Australia; l Kurongkurl Katitjin, Edith Cowan University, 2 Bradford St, Mount Lawley, 6050, Australia; m Gertner Institute for Epidemiology and Health Policy Research, Sheba Medical Center, 52126, Ramat Gan, Israel; n Epidemiology & Preventive Medicine Department, School of Public Health, Faculty of Medicine, Tel Aviv University, 69978, Tel Aviv, Israel; o South West Aboriginal Medical Service, 51/55 Forrest Avenue, 6230, Bunbury, Australia; p Academic Unit of General Practice, Australian National University, 0200, Canberra, Australia; q Population Health Program, QIMR Berghofer Medical Research Institute, 300 Herston Rd, 4006, Herston, Australia; r School of Public Health, University of Queensland, 266 Herston Rd, 4006, Herston, Australia; s Centre for Aboriginal Medical and Dental Health (CAMDH), Medical School, University of Western Australia, 35 Stirling Hwy, 6009, Crawley, Australia; t School of Population Health, Faculty of Medicine & Health, UNSW Sydney, 2052, Kensington, Australia; u Derbarl Yerrigan Health Service, 156 Wittenoom Street, East Perth, 6004, Australia

**Keywords:** Aboriginal/Torres Strait Islander peoples, Indigenous, Randomized controlled trial, Dementia, Blood pressure, Cardiovascular disease, Cognitive decline

## Abstract

**Background:**

Limited available data indicate that dementia prevalence rates among Aboriginal and Torres Strait Islander (hereafter Aboriginal) peoples are 3–5 times higher than the overall Australian population. Effective, pragmatic and scalable interventions are urgently required to address this disproportionate burden of dementia in Aboriginal populations.

**Methods:**

Kaat Koort is a pragmatic two-arm parallel-group randomized controlled trial which will recruit a sample of 354 participants from two Aboriginal community-controlled health services in the south-west of Western Australia. Eligible participants are aged 35–60 years with risk factors for cardiovascular disease. Participants will be randomized in a 1:1 ratio to receive either a 12-month multifactorial lifestyle intervention (guided by Aboriginal Health Practitioners) that involves cardiovascular risk management, a lifestyle program targeting diet and physical activity, and support for smoking cessation and depression, or usual care (control). The primary endpoints are change in (i) systolic, and (ii) diastolic blood pressure. Secondary endpoints are changes in other cardiovascular risk factors (elevated blood pressure, HDL cholesterol, HbA1c, waist circumference, and absolute cardiovascular risk score), cognitive functioning, and adherence to Australian dietary and physical activity guidelines. Outcomes will be collected at baseline, and 6- and 12-months post-baseline.

**Discussion:**

This trial aims to determine the efficacy of a multifactorial lifestyle intervention in reducing blood pressure among Aboriginal people aged 35–60 years at risk of dementia.

**Trial registration number:**

ACTRN12621001022853; Australian New Zealand Clinical Trial Registry identifier.

## 1 Background

The limited available data indicate that dementia prevalence among Aboriginal and Torres Strait Islander peoples (hereafter Aboriginal) are 3–5 times higher than the overall Australian population [1]. Data from New South Wales (NSW), Western Australia and the Northern Territory highlight that the age-standardised prevalence of dementia ranges from 21 to 28 % among men and women aged 60 years and older [[2], [3], [4]]. Alzheimer's disease is the most common type, accounting for 44 % of all dementia cases in urban and regional areas of NSW, followed by vascular dementia (17 %) [3].

The relationship between cardiometabolic health and brain health is well-understood and hence this study is named Kaat Koort—incorporating the Noongar language words for brain (Kaat) and heart (Koort). Modifiable risk factors for cognitive decline and dementia are similar to those for cancer and cardiovascular disease (CVD), including smoking, low physical activity, obesity, high blood pressure (BP), high cholesterol, and diabetes [5]. Additional risk factors include low education, depression, low cognitive engagement, hearing loss, low social contact, traumatic brain injury and air pollution [6]. Population attributable risks for the most common risk factors have been estimated globally [7] and for Australia [8,9]. Ashby-Mitchell et al. (2017) [8] estimated that the proportion of cases in Australia that could be prevented by removing modifiable risks was 17 % for midlife obesity, 18 % for physical inactivity, 4.3 % for smoking, 15 % for low education, 2.4 % for diabetes, 14 % for midlife hypertension, 8 % for depression, and 48 % for all risk factors combined [8]. These proportions pertain to the general Australian population, including but not specific to Aboriginal people. At the time of designing the study there were no equivalent studies specifically on Aboriginal populations; however, a recent report of potentially preventable dementia in a First Nations population in North Queensland found the highest dementia burden was attributed to hypertension (9.4 %), diabetes (9.0 %), obesity (8.0 %) and smoking (5.3 %) [9]. Given the higher rate of many risk factors in the Aboriginal population, successful reduction of these risk factors is likely to have an even greater impact on preventing and reducing the disproportionate burden of dementia among Aboriginal people.

Evidence is required to establish the feasibility, acceptability and efficacy/effectiveness of interventions to reduce cognitive decline and dementia in Aboriginal people. These must be informed by state-of-the-art knowledge about what works to prevent cognitive decline and dementia in other populations and what works for implementation in an Aboriginal context. Cognitive decline is of particular importance as it is an established precursor to dementia, supporting early intervention with the potential to preserve functionality.

Rather than address individual risk factors in isolation, randomized controlled trials (RCTs) in dementia prevention have adopted a multi-domain approach that targets multiple risk factors simultaneously to maximise the potential for risk reduction [[10], [11], [12], [13]]. Key to prevention of cognitive decline is the modification of risk factors through lifestyle changes and adherence to medications to improve cardiometabolic outcomes. Among community-dwelling individuals with conditions such as dementia and heart failure with long-term risk exposures such as hypertension, self-care maintenance behaviours were significantly lower in Aboriginal compared with non-Aboriginal individuals [14]. Implementation of behavioural change techniques are needed to achieve real changes in participant behaviour.

Recent reviews of cohort and experimental studies have indicated that several dietary patterns and nutritional components (e.g., Mediterranean diet, unsaturated fatty acids, vitamin D, vitamin B and antioxidants including vitamin E, vitamin C and flavonoids) are associated with significant cognitive benefits [15,16]. Such nutritional components may exert their protective function in multiple and convergent ways. The evidence for the protective effect of the Mediterranean diet against CVD and diabetes (which are precursors of/contributors to vascular cognitive decline) is well established [17]. However, the relationship with dementia is inconsistent [15,[17], [18], [19], [20]], likely due to differences in study populations, the definitions of the Mediterranean diet utilised, and experimental designs. There is a shift both in nutritional epidemiology and the neurosciences from a “single nutrient” approach to a “whole diet” paradigm, more broadly aimed at capturing the food synergy of the cumulative effects of overall diet patterns on the health status of the individual [15,18,21]. The Australian Dietary guidelines make recommendations based on whole foods and contain elements of a Mediterranean style diet, such as eating a diet high in vegetables, fruits, grains and cereals, moderate amounts of lean meats and alternatives, and reducing saturated fats, added salt and sugars, and alcohol [21].

The need for additional RCTs in different populations is widely agreed upon [15,[17], [18], [19], [20]]. Therefore, our aim is to examine the efficacy of a lifestyle modification program to improve cardiometabolic disease risk and prevent or delay the development of dementia in Aboriginal people with elevated risk for CVD onset or complications. We hypothesise that the adults in a dementia prevention intervention group will have a reduction in BP and cardiometabolic risk factors compared with usual care after a 12-month follow-up. Further, we anticipate that, compared with usual care, an intervention group will experience greater improvements in cognitive performance and significantly increased healthy diet and lifestyle behaviours.

## 2 Methods

### 2.1 Study design

**Fig. 1:**
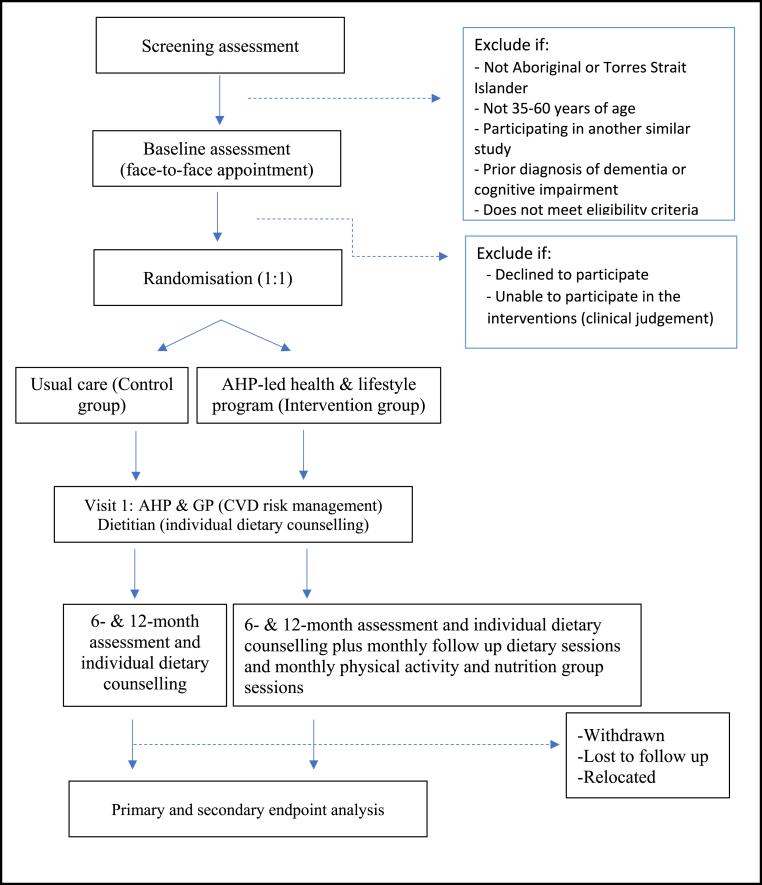
Study flow diagram from screening and baseline procedures to endpoint evaluation.

The Kaat Koort brain health study is a 1:1 pragmatic parallel-group individualised superiority RCT, testing the efficacy of a multifactorial, multidisciplinary Aboriginal Health Practitioner (AHP)-led health and lifestyle modification program in the intervention group to reduce BP and other secondary measures of cardiometabolic health compared with usual care in Aboriginal peoples. It will be implemented in two Aboriginal community-controlled health services in the south-west of Western Australia: South West Aboriginal Medical Service (SWAMS) in regional Bunbury and Derbarl Yerrigan Health Service (DYHS) in urban Perth. The study flow diagram for the 12-month intervention is shown in Fig. 1. All procedures will be performed in compliance with relevant laws and institutional guidelines. The final study protocol (Version 7; May 2024) was approved by the Western Australian Aboriginal Health Ethics Committee (HREC947).

### 2.2 Setting

SWAMS and DYHS were established by Noongar leaders and serve communities in Bunbury and Perth. The Bunbury and Perth regions have a population of almost 15,000 Aboriginal and Torres Strait Islander people aged 35 years and over [22]. Both regions overlap and are within a 2-hour drive so are easily accessible for a joint regional approach to dementia prevention.

### 2.3 Research governance

The overarching project steering committee is led by an Aboriginal researcher and supported by three other Aboriginal investigators and members of the Aboriginal Medical Service (AMS) organisations that are involved in the study. This includes the Chief Executive Officer of SWAMS and the Board of DYHS. The team make use of the high-quality infrastructure and expertise at their respective institutions to maintain Aboriginal governance in the project. Further, the team incorporates and supports partnership with the Aboriginal community-controlled health sector.

### 2.4 Study population

Participants are Aboriginal and Torres Strait Islander people residing in Bunbury and Perth in Western Australia, aged between 35 and 60 years (inclusive) and at high risk of CVD (see Section 2.6 for criteria). The selection of this age group aligns with the study's prevention focus and is supported by existing literature linking midlife cardiometabolic health to the risk of cognitive decline and dementia in later life.

### 2.5 Recruitment strategy

A broad recruitment strategy will be used to disseminate information about the study through yarning circles (a rigorous Indigenous qualitative research data gathering method [23]), Noongar radio and other information sharing activities conducted in partnership with DYHS and SWAMS. Flyers and advertisements will be placed in the SWAMS and DYHS clinics. Aboriginal Health Practitioners/nurses in daily contact with Aboriginal patients will identify potential eligible participants and inform them of the study.

### 2.6 Eligibility criteria

Participants aged 35–60 years from Bunbury and Perth at high risk of CVD (assessed during a screening visit; see criteria below) who are willing to provide written informed consent will be eligible for the study. Participants with a prior diagnosis of dementia and/or cognitive impairment (self-report), assessed as not being able to participate in the proposed intervention, or enrolled in another clinical trial are not eligible to participate. In addition, female participants who are pregnant or planning a pregnancy will also be excluded.

Participants are eligible if they satisfy one or more of the following criteria:

**Criteria 1**: Prior likely atherosclerotic CVD (patient self-report), including stroke, coronary artery disease (myocardial infarction, angina, previous revascularisation intervention), peripheral vascular disease and heart failure. People with atrial fibrillation (only) and valve disease (only).
**Criteria 2**: Current regular smokers (self-reported smoking status). Only tobacco smoking was included and those that smoke at least daily.
**Criteria 3**: Any two of the following risk factors (from previous health records within the previous 3 months) (see Table 1):

**Table 1:** Risk factors and cut-point values for inclusion in study (Criteria 3).

Risk factor	Cut-point value
Elevated BP	≥140/90 mmHg
Elevated waist circumference	Men: >104 cm
	Women: >88 cm
Reduced HDL-C	≤1.0 mmol/L
Elevated LDL-C	>2.0 mmol/L
Elevated HbA1c	>5.7 % or >39 mmol/mol

Participants who do not meet eligibility criteria 1 or 2 and are unsure if they meet eligibility criteria 3 will be asked to undertake a screening risk profiling visit to assess eligibility under criteria 3 (see section 2.9.1, below: *Screening for eligibility assessment*).

Interested participants are provided a verbal and/or written explanation of the study by the AHP/Registered Nurse (RN) in Plain Language Statements targeted at early high school (i.e. age 12–13 years) reading level. Eligibility for the study will be determined by the AHP/RN during a face-to-face visit. Once confirmed to be eligible, a visit will be organised for the baseline assessment. Informed consent procedures occur at the baseline visit. Participants will be informed of what procedures will occur in both the ‘usual care’ and ‘intervention’ groups.

### 2.7 Randomization, allocation concealment and blinding

Once participants are confirmed eligible and provide consent, they will be randomized 1:1 into either the usual care (control) or intervention group. The development of the randomization process will be carried out at The University of Melbourne. The randomization list will be computer-generated by an independent statistician and carried out centrally to ensure concealment. Stratified block permuted randomization will be used with the study site (Perth and Bunbury) and age group (35–44 years, 45–60 years) as stratification factors to ensure balance in treatment allocation within each study site. Study staff at both sites can access the randomization schedule using the primary (online) data collection and management tool, REDCap. An external committee will periodically check that the randomization protocol is maintained throughout the trial. Participants and study staff cannot be blinded due to the nature of the interventions. Study biostatisticians will remain blinded until the database has been cleaned, a blinded data review has taken place, and the database is ready for analysis. An overview of the study procedures is shown in Fig. 1.

### 2.8 Primary endpoints

1) Change in systolic BP (mmHg) from baseline to 12-months follow-up.
2) Change in diastolic BP (mmHg) from baseline to 12-months follow-up.

#### 2.8.1 Secondary endpoints

1) Change in proportion of participants with elevated BP (either systolic BP greater than or equal to 140 mmHg or diastolic BP greater than or equal to 90 mmHg) from baseline to 6- and 12-months follow-up.
2) Change in absolute cardiovascular risk score (calculated using age, gender, systolic BP, smoking status, total cholesterol, HDL-cholesterol and diabetes) from baseline to 6- and 12-months follow-up.
3) Change in waist circumference (cm) from baseline to 6- and 12-months follow-up.
4) Change in HDL-C (mmol/L) from baseline to 6- and 12-months follow-up.
5) Change in HbA1c (mmol/mol) from baseline to 6- and 12-months follow-up.
6) Change in cognitive function (measured using both the Symbol Digit Modalities Test and Cogstate Brief Battery) from baseline to 12-months follow-up.
7) Meeting the recommended Australian dietary and physical activity guidelines at 6- and 12-months follow-up.

Note: (a) Sitting BP will be measured in the brachial artery with a suitable cuff size using a calibrated Omron HEM-907 automated monitor (Omron Healthcare Co. Ltd., Kyoto, Japan) after at least 1 min of rest. Three measurements will be taken, separated by a 1-min interval; (b) There are many ways to calculate cardiovascular risk score. At the time of study design the National Vascular Disease Prevention Alliance (NVDPA) and Central Australian Rural Practitioners Association (CARPA) CVD risk assessment algorithm is used for Indigenous Australians [24]; (c) the Australian Dietary guidelines recommends consumption of five core foods groups and limiting intake of discretionary foods (containing saturated fats, added salt and sugars, and alcohol) [21]. A change in core food group intake and discretionary food intake from baseline to 6- and 12-months follow-up will be assessed using data from the digital Food Frequency Questionnaire (FFQ) [25] (see 2.12).

### 2.9 Procedures

#### 2.9.1 Screening for eligibility assessment

An AHP or RN at SWAMS/DYHS will complete a brief screening questionnaire with the participant to collect demographics and assess eligibility based on smoking status, history of CVD (angina, previous heart attack, peripheral vascular disease or stroke), heart failure, elevated BP, elevated waist circumference, reduced HDL-C, elevated LDL-C and elevated HbA1c. If the participant answers ‘no’ or ‘I don't know’, eligibility cannot be confirmed and a clinical assessment will be conducted (see 2.9.2A). These results will be used as baseline measures for those deemed eligible.

All individuals who meet the inclusion criteria will receive further advice and information about the RCT and will be invited to participate in the trial. All participants who consent to participate in the trial will be randomly allocated to intervention or usual care and followed according to protocols for these groups.

#### 2.9.2 Collection of baseline data

Baseline data will be collected via validated and reliable methods comprising self- or AHP/RN-administered questionnaires, and clinical assessment conducted by the AHP/RN. The following information will be collected from all participants in the intervention and usual care groups:

A. *Clinical assessment*: anthropometry (height, weight, BMI, waist and hip circumference); average BP (3 readings taken with digital BP machine); heart rate; lipids (total cholesterol, LDL-C, HDL-C, triglycerides), and HbA1c.
B. *General health and social demographic indicators*: medical history (atrial fibrillation/arrhythmia; kidney disease; Type 1 or 2 diabetes; sleep apnea; depression; arthritis; migraine; mental illness [other than depression]; liver disease [active, chronic or cirrhosis]; other serious condition[s]); medications, sleep [hours], family medical history; language spoken; marital status; education; work status, income; government benefits.
C. *General wellbeing*: Adapted Patient Health Questionnaire (aPHQ-9); Assessment of Quality of Life-4D (AQoL-4D) self-administered or interviewer.
D. *Cognitive assessment:* Cogstate Brief Battery (Cogstate) consists of four computerised tests of psychomotor speed, visual attention, visual learning, and memory and working memory; the SDMT assesses attention and processing speed. This assessment will be undertaken using a paper-based form with scores and forms uploaded to REDCap by the AHP/RN.
E. *Health behaviours*: diet, physical activity and alcohol intake collected from an Accredited Practising Dietitian (APD) via a digital FFQ that was adapted for use with Aboriginal adults [25].

### 2.10 Follow-up

Data will be collected at baseline, 6- and 12-months for both usual care (control) and intervention participants according to the schedule in Table 2.

**Table 2:** Study procedures and visits.

STUDY PROCEDURES	Screening^a^	Baseline	6 months	12 months
Anthropometry (AHP/RN objective assessment)	X^a^	X	X	X
Blood samples taken from venepuncture examined for: lipids, HbA1c (AHP/RN objective assessment)	X^a^	X	X	X
Systolic and diastolic blood pressure (AHP/RN objective assessment)	X^a^	X	X	X
General Health and Social Demographics Questionnaire		X	X	X
Patient Health Questionnaire-9 (aPHQ-9)		X	X	X
Assessment of Quality of Life (AQoL-4D)		X	X	X
I-ACE Food Frequency Questionnaire (FFQ)		X	X	X
Cognitive Function Assessment (Mixture of pen and paper and Laptop)		X		X
CVD and other risk management		X	X^b^	X^b^

a If these measurements are performed in the screening visit, they will then be used for the baseline assessment.

b intervention participants only.

### 2.11 Culturally appropriate measurement of cognitive function in Aboriginal people

There is a paucity of published literature evaluating common cognitive measures in Indigenous populations aged 60 years and under. A pilot study was conducted prior to the conduct of Kaat Koort to evaluate the performance and acceptability of cognitive measures among Aboriginal people aged 35–60 years of age. A range of measures were considered, including elements of the Trail Making Test [26], SDMT [27], Montreal Cognitive Assessment [28], and the Cogstate Brief Battery [29]. On balance, the SDMT and Cogstate Brief Battery were chosen for use in Kaat Koort given that participant scores were the closest to age expectations and qualitative feedback was consistently positive (unpublished data). The SDMT assesses attention and processing speed and is completed using a paper-based form. Cogstate consists of four computerised tests of psychomotor speed, visual attention, visual learning, and memory and working memory. Cogstate is portable, adaptable, is less associated with demographic variables such as education or language and has been used in Aboriginal populations and clinical trials [30]. Both the SDMT and Cogstate tests were reviewed with health service staff and other key informants to determine optimal methods of delivery and whether the tools required modification to ensure cultural safety, bearing in mind the need to retain measurement properties.

### 2.12 Electronic nutrition and physical activity assessment and monitoring tool adaptation

An 81-item FFQ was adapted for use with Aboriginal adults and will be administered via the digital Interactive Lifestyle Assessment, Counselling and Education (I-ACE) platform. Both the FFQ and I-ACE platform were developed by Gertner Institute in Israel for use in marginalised Indigenous populations with type 2 diabetes mellitus [31]. I-ACE was modified based on analysis of the 2012-13 National Aboriginal and Torres Strait Islander Nutrition and Physical Activity Survey (NATSINPAS) dataset to identify foods that contribute to greater than 80 % of nutrients and food groups of interest among non-remote Aboriginal people in Western Australia, and for sensitivity to measuring adherence to the Australian Dietary Guidelines [21]. Based on community feedback, the I-10.13039/100021131ACE food database was further modified to support a meal-based FFQ format with a main food and beverage list of 81 items and the ability to report on an additional >300 items on the digital platform [25]. All foods currently in the database were checked and harmonised with the nutrient values in AUSNUT 2011–2013. The embedded lifestyle educational materials were adapted based on the Australian Dietary Guidelines, and input from local healthcare providers and members of the target population. In addition, the graphics in the software (e.g., food pictures, infographics) were adapted for the local context [25].

### 2.13 Study treatment

#### 2.13.1 Intervention group

The AHP/RN-led intervention represents a collaboration between the participant and health care team. AHPs/RNs in partnership with the key coordinating general practitioners (GPs) from each clinic will coordinate the delivery of the intervention with the aim to complement services delivered through the main general practice clinic.

Key components include:

1. Baseline, 6- and 12-month clinical assessments as described in section 2.9.2.
2. *AHP/RN coordinated cardiometabolic risk factor management:* Nurses/AHPs will work with participants and primary care providers to coordinate care (including review and prescription of medication).
3. *Nutrition program:* The I-ACE platform will be used to conduct nutrition and physical activity assessments that are the basis for tailored individual lifestyle counselling. Study dietitians will receive training to utilise this program and conduct the counselling sessions. Individuals will participate in monthly individual counselling sessions and monthly group sessions with a dietitian over 12-months. Educational materials on diet (and physical activity) written in plain English and tailored to local Noongar cultural values will be given to participants to supplement face-to-face advice sessions. During group nutrition sessions participants will receive instruction on food label reading, meal planning, food purchasing, and simple cooking methods.
4. *Physical activity training:* Qualified exercise therapists have developed a physical activity program tailored for the age range, targeted at reducing cardiovascular risk factors and tailored to the specific needs of the participants. The exercise therapist will then train the AHP/RNs to support the delivery of the program to participants. Participants will be invited to attend two group sessions per month. The instructor will complete sessions built around muscle strengthening, aerobic exercise and each participant will receive a physical activity booklet (a culturally appropriate resource) with basic instructions on each exercise. The sessions and booklet will guide exercises that can be conducted outdoors (including walking) and at home (simple muscle strengthening activities). Participants will also be encouraged to adhere to the national guidelines of 150 min of physical activity per week.
5. *Smoking cessation*: Current smokers will be assessed on their readiness to cease smoking and will be provided appropriate counselling to encourage them to quit. Referrals to GPs in the clinics to facilitate prescribed smoking cessation medical therapy (e.g. varenicline, bupropion) or nicotine replacement therapy will also be offered.
6. *Depression*: The aPHQ-9 (which has been modified and validated for use in Aboriginal and Torres Strait Islander populations) will be completed and used to identify participants at risk of major depression (aPHQ-9 score >9). These participants will receive a referral and advice to see their GP for treatment. Individuals will be followed up at one-month intervals and supported to attend visits with their GP for management of depression by the study AHP/RNs.

#### 2.13.2 Usual care (control) group

1. Baseline, 6- and 12-month clinical assessments as described in section 2.9.2.
2. Nurses/AHPs will provide a cardiovascular disease risk management plan at baseline and coordinate with primary care providers.
3. Study dietitians will provide three individual dietary counselling sessions over 12-months (at baseline, 6- and 12-months) using the I-ACE platform, and offer standard dietary advice. Guided group or individual physical activity sessions will not be offered.
4. Participants at risk of major depression and those who indicate they are current regular smokers, will follow the existing usual care pathway of the health service—accordingly, AHP/RNs will refer them to a clinic GP for the purposes of (1) further treatment for potential mental health issues and (2) to support the participant to quit smoking.

### 2.14 Study power

**Fig. 2:**
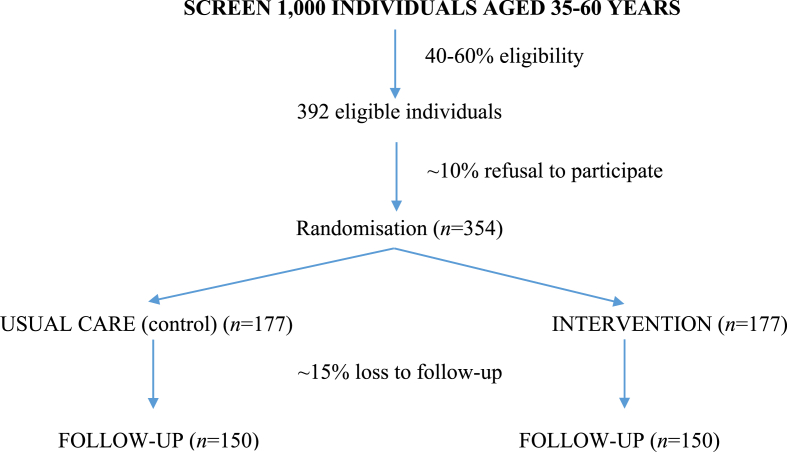
Flow chart.

A total sample size of 300 (150 control and 150 intervention subjects) provides 80 % power to detect a minimum clinically important difference of 4 mmHg in mean change in diastolic BP at 12 months follow-up between the intervention and control arm with two-sided alpha of 0.05 divided between the two primary outcomes. This assumes equal standard deviations of 11 mmHg in both groups based on estimates from Carrington et al. (2021) and no correlation between baseline and follow-up diastolic BP (conservative assumption) [32]. This sample size provides >95 % power to detect a minimum clinically important difference of 8 mmHg in the other primary outcome, systolic BP, between the two study arms, assuming a standard deviation of 15 mmHg [32]. Accounting for potential loss-to-follow up of 15 %, the trial requires 177 participants randomized in each group (see Fig. 2).

### 2.15 Data management

Data collection will be overseen by a Trial Manager and Trial Coordinator. All data will be stored securely on The University of Melbourne password protected server with data management based at The University of Melbourne. The majority of the data will be collected and stored on REDCap which is a secure server-based research data capture software accessible only by study researchers. Once data collection is complete, all data will be exported and stored on password protected folders on The University of Melbourne server.

Data collected via the I-ACE platform is stored on an Azure cloud server. The Azure platform includes industry-standard, built-in security and privacy features. I-ACE was developed and maintained throughout the study by Barillet (Israel) Ltd. The built-in data security features of I-ACE include separation of the application, business, and data layers of the program, a sophisticated permission system, and encryption (using SlowStart) of the identifying data of all users. Cumulative study participant data on dietary intake and physical activity will be downloaded from Azure platform by the Gertner Institute investigators, using only the participants’ study ID number, without other identifying data. Files will be prepared for data analysis at the Gertner Institute, and then securely transferred to The University of Melbourne study coordinators and stored securely on university servers.

Clinical data will be stored on Communicare (clinical data management system) so that participants can be followed up and reviewed by GPs and other health professionals at the trial clinics. Study-specific clinical items will be created in Communicare to ensure that relevant clinical data can be followed up.

Paper documents will be stored in a locked cabinet accessible only by the research team. Data sharing between organisations involved in the study is kept secure by clinical trial research agreements between the collaborating organisations. Upon completion of the study all participants will receive a summary of group results and study findings will be presented in publications and presentations.

### 2.16 Statistical analyses

A detailed statistical analysis plan will be finalised before locking the database. The analysis will be undertaken on an intention-to-treat basis by a biostatistician blinded to the group allocation from the Methods and Implementation Support for Clinical and Health research Hub at The University of Melbourne, with all randomized participants included according to the treatment they are randomized to receive.

Constrained longitudinal data analysis (cLDA) models will be used to examine the change in each of the primary outcomes (systolic and diastolic BP), with the response consisting of all scores (baseline, 6 months, 12 months) and the model including factors representing group (intervention/control), time, intervention by time interaction and the strata (site and age group), with the restriction of a common baseline mean across interventions. This is based on the assumption that at baseline there are no differences in the mean secondary outcomes between the treatment groups; namely, that the randomization is effective. The cLDA model provides valid inference if the missing data mechanism is missing at random. The mean change in each of systolic and diastolic BP from baseline to each follow-up time point between the two treatment arms will be obtained. The primary research question will be evaluated by obtaining the estimated differences between the two treatment arms in mean change in systolic and diastolic BP from baseline to 12-months follow-up (primary time point), two-sided 97.5 % confidence intervals and p-values. A similar approach will be used for continuous secondary outcomes. For binary outcomes, logistic regression models controlling for stratification factors will be fitted using generalised estimating equations to account for clustering of time within participants, with risk differences and 95 % confidence intervals calculated.

## 3 Discussion

### 3.1 Significance

An opportunity exists to build upon national quality improvement indicators for Aboriginal Community Controlled Health Sector (ACCHS) on absolute CVD risk assessment to support dementia prevention. Although CVD is largely preventable, there are unacceptably high rates in Aboriginal Australians, with hospitalisation rates for ischaemic heart disease at 25–44 years of age being 7–8 times higher compared with non-Aboriginal Australians [33]. Calabria et al. (2018) estimated that 16 % of Aboriginal people aged 35–74 years were at high risk of a primary CVD event and more than a quarter were at high absolute 5-year CVD risk, with the majority of those at high risk not receiving recommended evidence-based treatment [34]. Given the major synergies between CVD and dementia, an opportunity exists to provide pragmatic evidence-based strategies to support ACCHSs to meet CVD risk assessment guidelines, enhanced to include dementia and provide targeted lifestyle prevention and intervention programs for Aboriginal and Torres Strait Islander peoples at risk of cognitive decline and dementia.

### 3.2 Feasibility

This project brings together strong partnerships between Aboriginal primary health care services, The University of Melbourne, Curtin University, the Australian National University, Baker Heart and Diabetes Institute, Edith Cowan University and the Gertner Institute (Israel). The study will run in a region with almost 15,000 Aboriginal people aged 35 years and older and draw on the expertise of leading investigators with extensive experience in the conduct of complex RCTs with primary health care/communities. The collaborative teams bring together Aboriginal leaders in research and health service management, dementia prevention expertise, AHP/nurse-led interventions to reduce CVD risk, general practice research, biostatistics and psychology. The program has a rich research and clinical practice platform upon which to conduct this work towards dementia prevention in Aboriginal communities.

## CRediT authorship contribution statement

**Carrington CJ. Shepherd:** Writing – original draft, Supervision, Resources, Project administration, Methodology. **Melissa A. Dunham:** Writing – original draft, Supervision, Project administration, Methodology, Investigation. **Lina Gubhaju:** Writing – review & editing, Project administration, Methodology. **Karen E. Lamb:** Writing – original draft, Methodology. **Digsu N. Koye:** Writing – original draft, Methodology. **Phoebe Fitzpatrick:** Writing – original draft. **Emily Banks:** Writing – review & editing, Methodology, Funding acquisition. **Kaarin J. Anstey:** Writing – review & editing, Methodology, Funding acquisition. **Melinda Carrington:** Writing – review & editing, Methodology, Funding acquisition. **Daniel McAullay:** Writing – review & editing, Funding acquisition. **Ofra Kalter-Leibovici:** Writing – review & editing, Methodology, Funding acquisition. **Grace Joshy:** Writing – review & editing, Methodology, Funding acquisition. **Lesley Nelson:** Writing – review & editing, Supervision, Funding acquisition. **Jason Agostino:** Writing – review & editing, Funding acquisition. **Ellie Paige:** Writing – review & editing, Funding acquisition. **Kathleen Abu-Saad:** Writing – review & editing, Supervision, Methodology, Data curation. **Elise Alexander:** Writing – review & editing, Supervision, Project administration, Methodology. **Rona MacNiven:** Writing – review & editing, Methodology. **Kelsey Griffen:** Writing – review & editing, Methodology. **Fiona Collins:** Writing – review & editing, Methodology, Investigation. **Salena Linforth-Milham:** Writing – review & editing, Methodology, Investigation. **Dolores Gilbert:** Writing – review & editing, Methodology, Investigation. **Cindy Prior:** Writing – review & editing. **Sadia Rind:** Writing – review & editing, Investigation. **Richelle Douglas:** Writing – review & editing, Methodology. **Sandra Eades:** Writing – original draft, Supervision, Project administration, Methodology, Funding acquisition, Conceptualization.

## Ethics

The Western Australian Aboriginal Health Ethics Committee have provided the primary ethical approval (HREC947).

## Funding sources

The study was funded by a 10.13039/501100000925National Health and Medical Research Council grant (APP1151848), as part of a Targeted Call for Research into Dementia in Indigenous Australians. MJC receives an endowed fellowship in the Cardiology Centre of Excellence from Filippo and Maria Casella.

## Declaration of competing interest

The authors declare that they have no known competing financial interests or personal relationships that could have appeared to influence the work reported in this paper.

## Glossary

Aboriginal Health Practitioner: registered healthcare practitioners who provide clinical services and care with an emphasis on culturally safe practice for Aboriginal and Torres Strait Islander peoples
Kaat: Noongar language word for brain
Koort: Noongar language word for heart
